# Targeting Overconsumption of Sugar-Sweetened Beverages vs. Overall Poor Diet Quality for Cardiometabolic Diseases Risk Prevention: Place Your Bets!

**DOI:** 10.3390/nu9060600

**Published:** 2017-06-13

**Authors:** Benoit J. Arsenault, Benoît Lamarche, Jean-Pierre Després

**Affiliations:** 1 Centre de Recherche de l’Institut Universitaire de Cardiologie et de Pneumologie de Québec, Y-2110, Pavillon Marguerite D’Youville, 2725 Chemin Ste-Foy, Québec City, QC G1V 4G5, Canada; jean-pierre.despres@criucpq.ulaval.ca; 2 Department of Medicine, Faculty of Medicine, Université Laval, Québec City, QC G1V 0A6, Canada; 3 School of Nutrition, Université Laval, Québec City, QC G1V 0A6, Canada; Benoit.Lamarche@fsaa.ulaval.ca; 4 Department of Kinesiology, Faculty of Medicine, Université Laval, Québec City, QC G1V 0A6, Canada

**Keywords:** sugar-sweetened beverages, diet quality, abdominal obesity, type 2 diabetes, cardiovascular diseases

## Abstract

Chronic overconsumption of sugar-sweetened beverages (SSBs) is amongst the dietary factors most consistently found to be associated with obesity, type 2 diabetes (T2D) and cardiovascular disease (CVD) risk in large epidemiological studies. Intervention studies have shown that SSB overconsumption increases intra-abdominal obesity and ectopic lipid deposition in the liver, and also exacerbates cardiometabolic risk. Similar to the prevalence of obesity and T2D, national surveys of food consumption have shown that chronic overconsumption of SSBs is skyrocketing in many parts of the world, yet with marked heterogeneity across countries. SSB overconsumption is also particularly worrisome among children and adolescents. Although the relationships between SSB overconsumption and obesity, T2D, and CVD are rather consistent in epidemiological studies, it has also been shown that SSB overconsumption is part of an overall poor dietary pattern and is particularly prevalent among subgroups of the population with low socioeconomic status, thereby questioning the major focus on SSBs to target/prevent cardiometabolic diseases. Public health initiatives aimed specifically at decreasing SSB overconsumption will most likely be successful in influencing SSB consumption per se. However, comprehensive strategies targeting poor dietary patterns and aiming at improving global dietary quality are likely to have much more impact in addressing the unprecedented public health challenges that we are currently facing.

## 1. Introduction

The epidemics of obesity, type 2 diabetes (T2D), and cardiovascular diseases (CVD) are affecting most if not all developed countries around the world. While the prevalence of overweight, obesity, and T2D remain high in North America and Western Europe, obesity rates and T2D rates are increasing at a stunning pace in developing countries [1,2]. For instance, a recent study showed that in Mexico City inhabitants aged between 35 and 74 years, the excess mortality associated with previously diagnosed T2D accounted for one third of all deaths between 1998 to 2004 [3]. Added sugar is one of the most consistent dietary features found to be associated with obesity, T2D, and CVD rates in large epidemiological studies [4,5,6,7]. In some countries such as the Unites States, sugar-sweetened beverages (SSBs) account for almost half of the added sugar consumed nationally [8]. SSB consumption around the world has reached unprecedented proportions, and the rise in the prevalence of cardiometabolic risk factors in children such as abdominal obesity and insulin resistance has increased in parallel. SSBs typically include carbonated soft drinks, juice drinks (with added sugars), sports drinks, energy drinks, milkshakes, and iced tea or coffee. A recent modelling study performed by the Global Burden of Diseases Nutrition and Chronic Diseases Expert Group (NutriCoDE) estimated that up to 184,000 deaths per year could be attributed to the chronic overconsumption of SSBs [9]. Similar to the prevalence of obesity and T2D, studies analysing national surveys of food consumption have shown that the chronic overconsumption of SSBs is also skyrocketing in many parts of the world, yet with marked heterogeneity across countries [10].

Although the relationship between SSB overconsumption and obesity and T2D is quite consistent, whether this association reflects a causal link has been debated as some studies have shown that SSB consumption is closely associated with other factors such as poor overall diet quality as well as an unfavourable socioeconomic status. The objective of this review article is to present the latest research on the association between chronic overconsumption of SSBs, diet quality, and cardiometabolic diseases, and to present evidence arguing for and against the potential causal role of SSBs in the aetiology of cardiometabolic diseases.

## 2. Cardiometabolic Impact of Sugar-Sweetened Beverages Overconsumption

SSBs are the single greatest source of added sugars in most Western countries. SSBs are typically sweetened with high-fructose corn syrup (HFCS) or sucrose. Sucrose, also often referred to as table sugar, is a disaccharide composed of glucose and fructose linked via a glycoside bond. Most sucrose is obtained from sugar cane and sugar beets. Maersk et al. [11] showed in a six-month parallel intervention study of 47 overweight individuals that the consumption of 1 L/day of sucrose-sweetened beverages (cola) significantly increased visceral adipose tissue and hepatic fat accumulation compared to the consumption of 1 L/day of semi-skimmed milk, artificially-sweetened beverages (ASBs), or water. Although not associated with increases in body weight or total fat mass, the consumption of cola was linked with increases in plasma triglyceride and cholesterol levels. Interestingly, daily total energy intake did not appear to differ across subgroups, thereby suggesting that energy included in beverages could have been compensated for by reductions in energy from other sources. HFCS is produced by industrial processing of corn starch. It contains two monosaccharides, free fructose, and glucose in various proportions. Both fructose and glucose have different metabolic fates, an observation that has encouraged many to suggest that fructose may have a unique role in the pathogenesis of cardiometabolic diseases. This hypothesis has been supported by well-designed controlled studies. For instance, Stanhope et al. [12] enrolled 32 men and women who were either overweight or obese to a 10-week randomized clinical trial designed to determine the relative effects of glucose- or fructose-sweetened beverages on visceral adiposity, insulin sensitivity, and lipoprotein-lipid metabolism. During the intervention, study participants were fed an ad libitum diet with 25% of calories originating from glucose- or fructose-sweetened beverages. Although both diets increased body weight, only participants in the fructose group had increased visceral adipose tissue accumulation at the end of the trial. The area under the curve of insulin levels during a 3-h oral glucose tolerance test increased by 27% in the fructose group (significant) and by approximately 14% in the glucose group (nonsignificant). Similarly, 24-h post-prandial triglyceride and fasting apolipoprotein B levels, as well as small, dense low-density lipoprotein (LDL) levels and triglyceride levels in remnant-like particles were all increased in the fructose but not in the glucose group following the intervention. Kinetic studies with isotopic acetate infusions revealed that hepatic fractional de novo lipogenesis increased in the fructose but not in the glucose group, thereby providing evidence that fructose overconsumption could contribute to poor cardiometabolic health. Another investigation using a randomized parallel design by the same group revealed that the consumption of 10%, 17.5%, or 25% of total energy as fructose-sweetened beverages led to dose-dependent increases in plasma lipoprotein-lipid and uric acid levels in as little as two weeks in young adults [13]. Consumption of moderate (40 g/day) and high (80 g/day) amounts of fructose- and sucrose-sweetened beverages were also found to slightly (but significantly) decrease hepatic insulin sensitivity and increase LDL cholesterol levels (but not triglycerides) in healthy young men compared with similar amounts of glucose-sweetened beverages for three weeks [14]. However, studying the impact of four weeks of fructose- and glucose-sweetened beverages under hypercaloric diets (150 g/day), Silbernagel et al. [15] found no group differences with regards to visceral or hepatic fat accumulation, plasma insulin, or cholesterol levels between both study groups. Only triglyceride levels appeared to have increased following the fructose-based diet. Overall, most but not all of the above studies suggest that consumption of SSBs, which corresponds to or is slightly higher than a high SSB consumption in the general population, may be associated with visceral adiposity/ectopic lipid deposition, insulin resistance, and an impaired lipoprotein-lipid profile. However, it is stressed that most of these studies were performed over relatively short periods, by randomized trials that had limited sample sizes and have not all adequately accounted for concurrent changes in total calories intake.

## 3. Sugar-Sweetened Beverages Overconsumption and Cardiometabolic Diseases Risk

About a dozen large prospective epidemiological studies have documented the association between SSB consumption and the risk of cardiometabolic diseases such as obesity, metabolic syndrome, T2D, and CVD. This literature has recently been extensively reviewed by Malik and Hu [16]. In 2013, these investigators performed a meta-analysis that included 25,745 children and adolescents from 15 prospective studies and 174,252 adults from in seven prospective studies [17]. The results of this analysis suggest that a one serving per day increase in SSB is associated with a 0.06 unit increase in body mass index (BMI) per year in children and adolescent and with a 0.12 to 0.22 kg yearly weight gain in adults. Although this study clearly shows that a high consumption of SSBs might promote weight gain in children and adults, the impact of a moderate consumption of SSBs (between one serving per day and one serving per week) on long-term body weight changes could not be modelled. In addition to this report on weight gain, the same group also published evidence that SSBs overconsumption is linked with the onset of the metabolic syndrome (a constellation of CVD and T2D risk factors associated with abdominal obesity and insulin resistance) and T2D [7]. This meta-analysis included 310,819 participants and 15,043 cases of T2D from nine cohorts. Compared to individuals in the lowest SSB consumption quintile, those in the top quintile had a multivariable adjusted relative risk of developing T2D of 1.26 (95% confidence interval, 1.12–1.41). The analysis on the incidence of metabolic syndrome included 19,431 participants and 5803 incident cases of metabolic syndrome from three cohorts. Compared to individuals in the lowest SSB consumption quintile, those in the top quintile had a multivariable adjusted relative risk of developing the metabolic syndrome of 1.20 (95% confidence interval, 1.02–1.42). Shortly after, investigators of the PREDIMED trial reported similar findings [18]. In this cohort, overconsumption of SSBs (≥5 servings per week vs. 0 servings per week) was positively associated with the incidence of the metabolic syndrome after multivariable adjustment (hazard ratio = 1.43 (95% confidence interval, 1.00–2.15)) while a moderate consumption (1–5 servings per week) was not (hazard ratio = 0.91 (95% confidence interval, 0.74–1.12)). Interestingly, the prevalence of individuals reporting consuming ≥5 SSB servings per week in this Mediterranean population ranged between 5% and 10%. Individuals reporting consuming ≥5 SSB servings per week were also less likely to adhere to the Mediterranean diet and consume less fruit, while being more likely to consume baked products, alcohol, and a higher total daily energy compared to other study participants. Another recent meta-analysis by Imamura et al. [4] revealed that higher consumption of SSBs was associated with a higher T2D risk (18% increase in risk per serving per day), independently of adiposity. This analysis, which was based on 38,253 cases of T2D, also revealed that ASBs as well as fruit juice consumption could be linked with T2D incidence, although the risk estimates were not as high compared to SSBs and the associations were likely to involve bias according to the authors. In an effort to further support the link between SSB consumption and non-alcoholic fatty liver disease (NAFLD), Wijarnpreecha et al. performed a meta-analysis of seven (mostly cross-sectional) studies and reported that individuals consuming SSBs were at increased risk of NAFLD compared to those not reporting consuming SSBs (hazard ratio = 1.53 (95% confidence interval, 1.34–1.75)) [19].

Similar measures of associations were recently reported for CVD incidence in the meta-analysis of Narain et al. [20]. This study included results from 308,420 individuals from seven prospective cohort studies. Compared to individuals with a low SSB consumption (usually in lowest quartiles or quintiles), those who reported the highest SSB consumption (usually in the top quartiles or quintiles) had a relative risk of 1.10 (95% confidence interval, 1.02–1.18) for total cardiovascular events. Interestingly, the risk associated with an elevated consumption of SSB was higher for myocardial infarction (hazard ratio = 1.19 (95% confidence interval, 1.09–1.31)) compared to stroke (hazard ratio = 1.10 (95% confidence interval, 0.97–1.25)), other vascular events (hazard ratio = 1.09 (95% confidence interval, 0.82–1.45)), or mortality (hazard ratio = 1.03 (95% confidence interval, 0.91–1.18)). Other meta-analyses also reported small but significant associations between SSB consumption and the risk of hypertension [21] as well as the risk of chronic kidney disease [22].

The above studies have established a clear association between SSB consumption and a broad range of cardiometabolic diseases. However, it is worth highlighting that these studies share the well-known limitations of observational studies. For instance, although some consideration is given to potential confounding factors (age, smoking, socioeconomic status, cardiometabolic risk factors, total energy intake, alcohol consumption, intake of other foods, etc.) in the regression models, the issue of residual confounding cannot be fully addressed, which could lead to an inaccurate estimation of the reported measures of associations. Many confounders are often imprecisely measured in large datasets and other confounders are often not even measured or considered. A proper assessment of physical activity/sedentarity is also lacking in most studies. It is also worth highlighting that few “dose-response” associations with the risk of cardiometabolic diseases have been reported. For instance, the results of the majority of observational studies suggest that individuals in the top quartiles or quintiles of SSB consumption are at elevated risk of obesity, T2D, or CVD, while individuals in other groups may not have been at increased risk, thereby highlighting that SSB overconsumption may be potentially harmful while a moderate consumption (less than one per day but more than one per week) may not be. Additionally, although results from the above studies consistently suggest that individuals in the highest SSB consumption categories may be at risk, with elevations in risk ranging from 10% to 30%, this level of risk could be qualified as at best modest.

## 4. Is Tt the Sugar-Sweetened Beverages per Se or Are They Partners in Crime?

Few studies have directly compared the impact of SSB consumption to other foods regarding the risk of developing cardiometabolic diseases over long periods of time. One of these studies revealed that each daily SSB serving was associated with a one-pound increase in body weight over a four-year period after adjusting for age, baseline BMI, and other potential confounders including many dietary items in a pooled analysis from the Nurses’ Health Study (I and II) and the Health Professionals Follow-up Study [23]. This study also revealed that the dietary items that were associated with the highest weigh gain over the same period were French fries (3.35 lb), potato chips (1.69 lb), potatoes (1.28 lb), as well as unprocessed and processed red meats (0.95 and 0.93 lbs, respectively). Interestingly, food items found to be negatively associated with weight gain included yoghurt (−0.82 lb), nuts (−0.57 lb), fruits (−0.49 lb), whole grains (−0.37 lb), and vegetables (−0.22 lb). It is interesting to note that many of the foods found to be negatively associated with weight gain contained various amounts of sugar, thereby highlighting that sugar consumption per se may be negatively or positively associated with weight gain, depending on its food source. In a recent modelling analysis based on the National Health and Nutrition Examination Surveys and several of the above-stated meta-analyses, Micha et al. [24] reported that the over- or the under-consumption of 10 dietary factors could explain up to 45% of cardiometabolic deaths (from coronary heart disease, stroke, or T2D). Dietary factors positively linked with cardiometabolic deaths included sodium, processed and unprocessed meats, and SSBs, while factors negatively associated with cardiometabolic deaths include polyunsaturated fats, nuts and seeds, whole grains, fruits, vegetables, and seafood omega-3 fats.

SSBs, fried foods, processed and unprocessed red meats, refined grains, snacks, and desserts are all part of the so-called Western diet. People who adhere to this pattern also tend to eat more calories per day compared to those who adhere to a prudent dietary pattern, a pattern associated with a higher overall dietary quality that is recommended in most dietary guidelines around the world. Interestingly, individuals who report adhering to a Western dietary pattern also tend to report eating out of home more often. This dietary pattern is also negatively correlated with socioeconomic status and is increasingly observed in developing countries [25], which is particularly worrisome given its strong association with the long-term risk of T2D and CVD [26,27].

One of the reasons why many public health advocates rapidly point the finger at SSBs to identify them as the main (and in some cases the only) driver of cardiometabolic disease is based on the fact that SSBs, unlike other constituents of the Western dietary pattern, provide a significant amount of extra or “unnecessary” calories to the human body as these calories do not contribute to satiety and may even promote overeating [28]. Those calories consumed on a regular basis do contribute to a positive energy balance and long-term weight gain and cardiometabolic risk. Whether the weight gain associated with SSB consumption is the result of more added sugars to one’s diet or whether simply the extra calories from SSBs contribute the weight gain is still under debate, and it is beyond our objective to address this issue as others have recently thoroughly addressed this timely topic [29,30,31].

In order to determine whether SSB consumption is an actionable risk factor that should be targeted for the prevention and/or management of cardiometabolic diseases, the potential confounding factors mediating the relationship between SSBs and the risk of cardiometabolic diseases need to be considered. This is best illustrated by the Kangbuk Samsung Health Study, which has recently documented the relationship between SSB consumption and coronary atherosclerosis measured by computed tomography in a cross-sectional study that included 22,210 men and women with food frequency questionnaire (FFQ) data available [32]. In this study, compared to individuals who reported never consuming sugar-sweetened carbonated drinks, those who reported consuming ≥5 per week had an odds ratio for the presence of coronary artery calcium of 1.31 (95% confidence interval, 1.06–1.62). Also in this study, those who reported drinking between 3 and 5 sugar-sweetened carbonated drinks per week did not have a higher risk for the presence of coronary artery calcium (odds ratio = 0.88 (95% confidence interval, 0.72–1.07)), a finding that further highlights the notion that moderate SSB consumption may not cause atherosclerotic CVD. Interestingly, compared to individuals who reported never consuming sugar-sweetened carbonated drinks, those who reported consuming ≥5 per week ate approximately 29% more calories per day, 52% more red and processed meat, and 60% more sugary foods. These individuals were also twice as likely to be smokers. Adjusting for some of these risk factors attenuated the association between SSB consumption and the presence of coronary artery calcium. The same is true for the variables that may predict SSB consumption.

**Figure 1:**
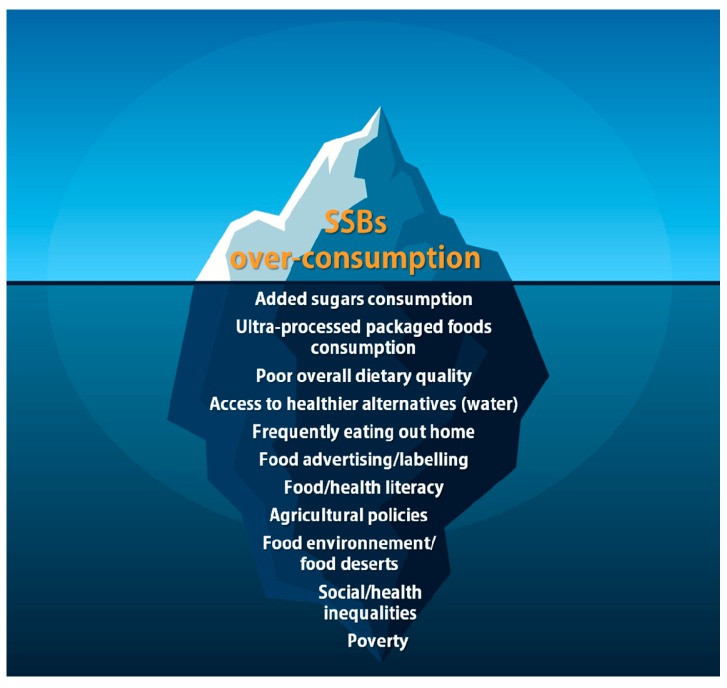
Schematic representation of factors associated with sugar-sweetened beverage (SSB) consumption.

In this regard, it has been known for decades that most dietary habits are formed during early childhood, and the same holds true for SSB consumption. A recent systematic review of the literature published aimed at identifying the determinants of SSB consumption in children under 7 years of age. Reviewed articles included reports from intervention, prospective, and cross-sectional studies. Many factors associated with elevated SSB consumption in children were identified such as child’s preference for SSBs, child television viewing/screen time, child snack consumption, parents’ lower socioeconomic status, parents’ younger age, parents’ SSB consumption, formula milk feeding, early introduction of solids, using food as rewards, parental-perceived barriers, attending out-of-home care, and living near a fast-food/convenience store. Additional factors were associated with lower SSB consumption in children, such as parental positive modelling, parents being married/cohabiting, school nutrition policy, and living near a supermarket. Results of this study suggest that drivers of SSB consumption are multifactorial. It also suggests that there are strong interrelationships among these determinants and that targeting only one of them in isolation without the others might not be effective in reducing SSB consumption in children or at the population level, although this still needs to be demonstrated. In this regard, a recent study confirmed a significant decline in added sugars consumption and availability in Australia between 1995 and 2011–2012 using four independent datasets without significant declines in obesity rates. Figure 1 presents a schematic representation of the correlates of SSB consumption, confounding factors that mediate the relationship between SSB consumption and the risk of cardiometabolic diseases, as well as the factors that predict SSB consumption, overall highlighting the notion that a comprehensive set of barriers and facilitators will need to be considered if we aim to reduce SSB consumption at the population level.

## 5. Disparities in Sugar-Sweetened Beverage Overconsumption around the World

Almost all the studies presented and discussed above were performed in homogeneous economically developed countries. While the evidence suggests that the deleterious impact of chronic overconsumption of SSB is independent of ethnic background, population-wide levels of SSB consumption are extremely heterogeneous across countries and could explain why some countries may be more likely than other to witness changes in cardiometabolic diseases incidence compared to others over the next few years. In this regard, the NutriCoDE study group [33] recently presented data on SSB consumption across different age ranges and sex from 51 countries representing 63% of the world’s population based on national or sub-national diet surveys for the year 2010. Results of this study suggest that regions of the world with the highest consumption of SSBs included Latin America, the Caribbean, and North America, while the lowest consumption is recorded in East Asia. In some parts of Latin America, the average SSB consumption exceeded 3 servings/day. Popkin and Hawkes [34] recently investigated the 2009–2014 changes in SSB sales in different regions of the world using data from the Euromonitor Passport International Database. Their investigation revealed that SSB sales increased in most low-income and middle-income countries, with Chile being the country with the most important rise in SSB sales during this period. Interestingly, they showed that SSB sales decreased in many high-income regions such as North America, Australasia, and Western Europe, although they remain on average very high compared to other countries. Considering the uncertainties with regard to the potential independent impact of SSBs on obesity levels, this observation suggests that consumers are probably more aware of the detrimental effects of chronic overconsumption of SSBs (despite them being heavily marketed compared to healthier alternatives). Under the hypothesis that SSB consumption is a strong and independent determinant of obesity, such a massive reduction in SSB sales observed at nation-wide levels should be associated with decreases in overweight/obesity. However, the prevalence of overweight/obesity has not decreased over the same period; rather it has increased significantly.

**Figure 2:**
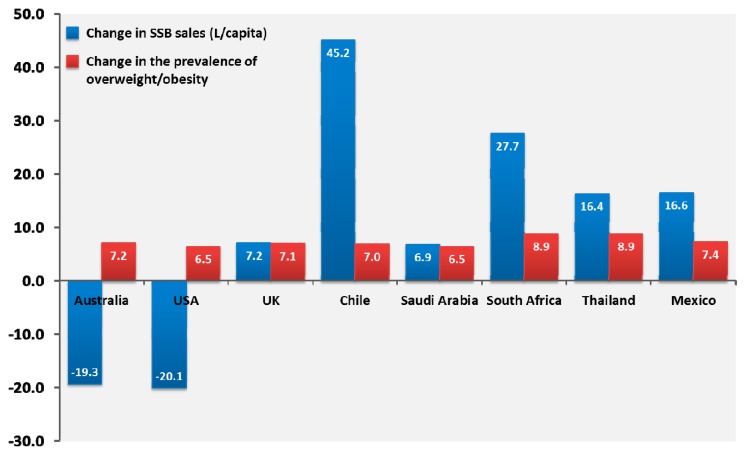
2002–2014 changes in sugar-sweetened beverage sales presented in litres per capita, and 2002–2014 changes in the percentage of the population who is either overweight or obese (body mass index ≥ 25 kg/m^2^) in selected countries from various regions of the world. Sugar-sweetened beverages were defined as the sum of on trade (bars, cafés, restaurants, etc.) and off trade (grocery stores, independent retailers, etc.) sales in any sugar-sweetened sodas (regular cola carbonates, lemonade/lime carbonates, ginger ale, tonic water, and orange carbonates), juice drinks (up to 24% juice), and nectars (25–99% juice), as well as sports drinks and energy drinks. Low-calorie cola carbonates and 100% fruit juices were not included. Data on body mass index changes were obtained from the World Health Organisation website.

To further illustrate this point, we have also turned to the Euromonitor International Passport Database and obtained SSB sales (soft drinks, juice drinks, energy drinks, and sports drinks) between 2002 and 2014 together with the prevalence of overweight/obesity in selected countries across most continents (from the World Health Organisation). Our results, presented in Figure 2, show that among countries of different continents with heterogeneous populations and socioeconomic statuses, the country-level SSB sales are very heterogeneous. In Western populations such as in the Unites States and Australia, SSB sales have significantly decreased, while they have increased significantly in other countries such as the United Kingdom, Chile, Saudi Arabia, South Africa, Thailand, and Mexico. Parallel to these changes, the percentage of individuals who were considered to be overweight or obese (BMI ≥ 25 kg/m^2^) increased everywhere, regardless of country-level SSB sales. This “pragmatic” observation also highlights the point that targeting SSBs in isolation without other efforts to improve overall nutritional quality may not lead to lasting changes in the prevalence of overweight/obesity.

As previously stated, SSB consumption is strongly associated with many confounding factors such as a Western type diet, ultra-processed food consumption, overall poor diet quality, smoking, less exercise, etc., which has led many to suggest that SSBs may also be a marker of poor lifestyle habits rather that an actionable risk factor for cardiometabolic diseases. Although cross-sectional studies have suggested that SSB overconsumption is correlated with poor diet quality [35], one argument against SSBs being a marker of nutritional quality is that national SSB sales/consumption appears to be decreasing in the absence of meaningful changes in nutritional quality [36]. A recent study also showed that a six-month intervention aiming at decreasing SSB consumption in a sample of adults with a high prevalence of obesity was not linked with improvements in other components of the diet (except vegetables consumption, which increased significantly from baseline) [37].

In developing countries, however, the rise in SSB sales/consumption appears to increase in parallel to the rise in ultra-processed food consumption [38,39]. Altogether, these observations suggest that the Western population seems to be increasingly aware of the detrimental impact of SSB overconsumption, and the consumption of SSBs appears to be increasingly “denormalised.” As countries in North America and Western Europe where SSB consumption appeared to be decreasing only accounts for about 10–20% of the world population at best, these gains will eventually need to be applied to the rest of the world and, more importantly, be coupled with improvements in overall dietary quality to address the epidemics of cardiometabolic disease.

## 6. Impact of Decreasing Sugar-Sweetened Beverage Intake

Although decreases in SSB consumption may not correlate with reductions in obesity rates at the national level, several studies have shown in intervention trials that decreasing SSB consumption may be associated with cardiometabolic benefits. In 2012, de Ruyter et al. [40] showed that masked replacement of SSBs with ASBs reduced weight gain in healthy Dutch children. This school-based intervention trial included 641 normal weight children aged between 5 and 12 years of age, randomized to receive 250 mL of SSBs (104 calories) or 250 mL of ASBs (0 calories) per day for 18 months. During the trial, children randomized to SSBs gained 7.37 kg on average, while children randomized to ASBs gained 6.35 kg on average. In the same issue of the New England Journal of Medicine, Ebbeling et al. [41] reported the results of another SSB reduction randomized clinical trial conducted in adolescents. This trial included 224 adolescents who were either overweight or obese who reported consuming at least one serving of SSBs or 100% fruit juice per day, and was designed to last two years. During the first year, adolescents randomized to the experimental group were advised to stop drinking SSBs as part of a multifactorial intervention during which water and ASBs were provided and were followed during the second year without intervention. The primary outcome of the study was change in BMI at two years. The intervention was very successful at reducing SSB consumption. At one year, adolescents included in the control group gained weight while those included in the intervention did not. However, changes in BMI at two years were comparable between the intervention and control groups. This suggests that intervention targeting SSB consumption in adolescents may not be successful if they are not sustained over long periods of time. Another study conducted in 1140 Brazilian fourth graders revealed that an education program aimed at discouraging children to drink SSBs during a full school year led to significant changes in SSB consumption (−56 mL per day on average) while having no effect on BMI, even in children who were characterized by excess body weight at baseline [42].

Altogether, these findings suggest that interventions aimed at reducing SSB intake on body weight have yielded conflicting and inconsistent results. However, given the somewhat moderate association between BMI and the risk of T2D and CVD as well as the results of recent studies that have shown that a more refined assessment of body composition or body fat distribution such as visceral fat or hepatic fat accumulation [43,44], measuring or judging the efficacy of SSB reductions by their effect on total body weight could be misleading. In this regard, several cross-sectional studies have shown that individuals who report consuming SSBs on a daily basis carry more visceral and liver fat accumulation, regardless of body weight [45,46,47,48]. In the Framingham Heart Study, determining the associations between SSB consumption on six-year changes in visceral adipose tissue accumulation, Ma et al. [49] reported that individuals who reported consuming SSBs on a daily basis gained approximately 30% more visceral fat compared to individuals who did not consume SSBs, even after adjustment for potential confounders including changes in body weight. Further, Campos et al. [50] recently showed in a randomized clinical trial that included 27 men with a BMI greater than 25 kg/m^2^ with a high consumption of SSBs that substituting SSBs with ASBs was associated with a 74% reduction of intra-hepatic lipids (measured by magnetic resonance spectroscopy) in just 12 weeks. At the end of the trial, participants randomized to continue their habitual consumption of SSBs were consuming more than 600 kCal/day on average compared to participants randomized to ASBs. Although an important effect on liver fat accumulation was noted, body weight, visceral adiposity, insulin sensitivity, and (unexpectedly) post-prandial triglyceride levels did not improve in the 12 weeks following this substitution.

Results of the above studies suggest that assessing key components of body fat distribution such as visceral and hepatic fat may prove to be crucial to properly evaluate the effect of SSB reduction on cardiometabolic health. Additionally, given the notion that a poor overall dietary quality is likely to contribute more to cardiometabolic disease risk than individual dietary factors such as SSBs [24], it is unclear how targeting only SSBs without improving overall dietary quality will prove to be beneficial. In this regard, studies are urgently needed to determine whether targeting SSBs, with or without parallel improvements in overall dietary quality, could influence body fat distribution and cardiometabolic health in order to determine whether targeting SSB intake in isolation or in conjunction with other changes aiming at improving dietary quality will result in lasting changes in cardiometabolic health in children, adolescents, and young adults (who are the greatest consumers of SSBs) [33].

## 7. Conclusions

Obesity and T2D rates are increasing in almost all jurisdictions around the world and will likely cause prejudice to improvements in cardiovascular mortality rates that we have seen decline since the 1970s. Obesity and T2D are complex diseases that are explained by a variety of factors, some related to food consumption (high energy intake, poor dietary quality/ultra-processed foods consumption, food marketing, food availability, culture, etc.) and some less related to diet such as genetic and epigenetic factors, neurobiological factors, adipose tissue function, gut microbiota, lack of physical activity/exercise, screen and sitting time, the built environment, lack of sleep, air pollution, and socioeconomic status, etc. Such a complex interplay of factors make it unlikely that putting the focus on one macronutrient (sugar) or one food item (SSBs) will solve the obesity and T2D epidemics. Nevertheless, the chronic overconsumption of SSBs is associated with a broad range of cardiometabolic diseases such as abdominal obesity, hepatic fat accumulation, metabolic syndrome, T2D, and CVD. Although individuals who consume small quantities or no SSBs at all have a better prognosis than individuals who consume SSBs on a daily basis, it is unsure whether SSBs alone are a driver of poor cardiometabolic health or merely a marker of an overall poor dietary quality or even of a lower socioeconomic status associated with high risk lifestyle habits. Therefore, interventions aimed at targeting overall dietary quality and the determinants of SSB consumption are more likely to lead to lasting changes in SSB consumption and to, overall, more substantial improvements in cardiometabolic health. It is encouraging to note that both SSB consumption and the vast majority of the determinants of SSB consumption are modifiable. However, in order to modify them, unprecedented interventions targeting children directly, their parents, as well as their school, workplace, and built environment will likely be required if we want to be serious in our efforts to reduce SSB consumption, improve dietary quality, and ultimately reduce the risk of cardiometabolic diseases at the population level.
